# Supplementary material for: A set of multi-entry identification keys to African frugivorous flies (Diptera, Tephritidae)
Source: Zookeys. 2014 Jul 24;(428):97–108. doi: 10.3897/zookeys.428.7366 (PMC4143993; doi:10.3897/zookeys.428.7366)
Supplement: Supplementary material 6 — Key to Ceratitis [file zookeys-428-097-s006.zip › SF6_ZooKeys_key to Ceratitis/key/SF6_ZooKeys_key to Ceratitis/Media/Html/Ceratitis aliena.htm]

Ceratitis (Ceratalaspis) aliena (Bezzi)


***Ceratitis*** **(*Ceratalaspis*)
*aliena* (Bezzi)**

*Pardalaspis aliena* Bezzi, 1920: 231.

Body
length: 4.02 (3.40-4.50) mm; wing length: 3.96 (3.65-4.35) mm.

Male

Head.
Antenna yellow to yellow-orange. Third antennal segment twice as long as second
segment. Arista with short hairs over entire length. Frons with short scattered
hairs which are largely the same colour as frons; convex, in lateral view not
distinctly projecting forwards at antennal implant. Chaetotaxy normal for
subgenus.

Thorax.
Postpronotum white, unspotted. Ground colour of mesonotum pale with orange
tinge; spots pattern variable, sometimes spots confluent forming dark lateral
stripes (in that case there are usually interrupted anteriorly of transverse
suture). Chaetotaxy normal for subgenus. Scapular setae black, inner ones
sometimes pale. One anepisternal bristle. Scutellum white basally, otherwise
yellow with three black separate markings apically, extending at most till
halfway dorsal surface, sometimes limited to apical margin; basally without dark
spots, only slight darker colouration. Subscutellum completely dark.

Legs.
Yellow; setation typical for subgenus, mainly pale, especially on femora.
Posterior and posterodorsal rows on fore femur pale.

Wing.
Bands with yellow-brown markings. Banding, marginal band with clear
interruption after cell R1; cubital and medial bands joined with marginal band;
crossvein r-m before middle of discal cell. Crossvein dm-cu posteriorly more
inwards than anteriorly. Extension cup sometimes weakly sinoid.

Abdomen.
Pale yellow or more brownish. Setation and banding typical for subgenus.

Female

As
male except for the following characters: Crossvein r-m rarely at middle.
Oviscape shorter than abdominal terga 3-6 combined combined.

(Description
after De Meyer, 1998)
